# Structured-Light Based 3D Reconstruction System for Cultural Relic Packaging

**DOI:** 10.3390/s18092981

**Published:** 2018-09-06

**Authors:** Limei Song, Xinyao Li, Yan-gang Yang, Xinjun Zhu, Qinghua Guo, Hui Liu

**Affiliations:** 1 Key Laboratory of Advanced Electrical Engineering and Energy Technology, Tianjin Polytechnic University, Tianjin 300387, China; songlimei@tjpu.edu.cn (L.S.); lixinyao211314@gmail.com (X.L.); xinjunzhu@tjpu.edu.cn (X.Z.); 2 School of Mechanical Engineering, Tianjin University of Technology and Education, Tianjin 300222, China; Yan_gangYang@163.com; 3 School of Electrical, Computer and Tele communications Engineering, University of Wollongong, Wollongong NSW2500, Australia; 4 China packaging & Test Center, Tianjin 300457, China; liuhui@packagetest.net

**Keywords:** structured light, cultural relics packaging, 3D reconstruction

## Abstract

The non-contact three-dimensional measurement and reconstruction techniques have played a significant role in the packaging and transportation of precious cultural relics. This paper develops a structured light based three-dimensional measurement system, with a low-cost for cultural relics packaging. The structured light based system performs rapid measurements and generates 3D point cloud data, which is then denoised, registered and merged to achieve accurate 3D reconstruction for cultural relics. The multi-frequency heterodyne method and the method in this paper are compared. It is shown that the relative accuracy of the proposed low-cost system can reach a level of 1/1000. The high efficiency of the system is demonstrated through experimental results.

## 1. Introduction

Cultural relics are precious due to their historical, artistic, scientific, and social values. The exchange of precious cultural relics has also become increasingly frequent [1,2]. The fragile cultural relics need to be packaged carefully during transportation [3]. Three-dimensional scanning technology is promising for cultural relic packaging. The establishment of high-precision and realistic 3D models of cultural relics is of great significance in digital museums, conservation and research of cultural relics, appreciation and display of cultural relics [4]. Manually produced 3D models can be used to produce packaging solutions in a simple and time-saving manner. However, there are still some problems to be solved to achieve fast, accurate, realistic, and low-cost three-dimensional measurements and reconstructions of cultural relics [5,6,7,8].

At present, the commonly used 3D measurement methods for digital reconstruction of cultural relics include laser scanning and close-range photogrammetry [9,10,11,12,13,14,15,16]. In 2012, Rodríguez-Navarro carried out a test on a small stone sculpture and an architectural element and compared the results from a triangulation-based Nextengine laser scanner and a point cloud generated by AGISOFT Photoscan [9], which was obtained with less effort using photogrammetry. The comparison was later extended by Remondino [10], evidencing the non-negligible influence of the SFM (Structure-From-Motion) algorithm and Image Matching (IM) algorithms on the final results. However, as demonstrated in digitizing an entire museum [11], the efficiency of image-based 3D digitizing of small objects is clear, but the metrological quality of many 3D data, although comparable to or better than TOF (Time-Of-Flight, manufactured by STMicroelectronics, Munich, Germany) and PS (Pulse Sensor) (manufactured by Triple-IN, Hamburg, Germany) laser scanners, does not seem always at the same level of high-end triangulation-based range devices. Wu used an Inspeck color three-dimensional scanning system to scan the head of cultural relics from the Terracotta Army so that cultural relic models and texture information could be obtained [12]. However, the cost of the equipment is very high, e.g., the price of a single camera is $8000–13,000, and the edge data is not accurate enough. Xu used Leica’s HDS8800 3D laser scanner (Leica Geosystems, Heerbrugg, Switzerland) to obtain three-dimensional heritage models [13] and the cost of this equipment is also a concern. Accurate point cloud data of the surfaces of cultural relics can be obtained by laser scanning, but some difficulties are encountered in the point cloud data splicing for relatively complex areas of the scanned object. Sometimes manual mark points are needed, which may not be allowed for cultural relics [14,15,16,17,18]. For objects with darker colors, the low reflectivity will affect the scanning quality. In addition, the cost of laser scanning is extremely high and there are also deficiencies in the protection of cultural relics. Close-range photogrammetry technology was first applied to building surveys. Most of the domestic applications were focused on the archaeological surveys of cultural relics. However, the work in the field heavily relies on a series of precautions, safeguards and redundancy strategies [19,20,21,22].

The optical projection method, with Gray code and phase shift, is commonly adopted in the 3D measurement products of many companies [23], such as the Atos-I type structured light three-dimensional measurement system of GOM company (Braunschweig, Germany), the COMMET series of structured light three-dimensional measurement systems of Steinbichler company (Neubeuern, Germany); the optoTOP series of structured light three-dimensional measurement systems of Breuckmann company (Heiligenhaus, Germany); the OKIO-II type three-dimensional scanner of Beijing Tianyuan three-dimensional technology Co. Ltd. (Beijing, China); the three-dimensional scanner of Shanghai Digital Technology Co. Ltd. (Shanghai, China,); and the CPOS three-dimensional scanner of Tianjin Century Power Photoelectric Science Instrument Co., Ltd (Tianjin, China). As the encoding method of Gray code mainly depends on the binarization of the image to be encoded, it is generally necessary to spray developer to achieve better measurement performance for situations where the color of the object surface changes rapidly, or the object in the scene is in a different brightness.

Zheng used a three-dimensional measurement method for the measurement and reconstruction of collections of artifacts based on structured light technology, but the measurement procedure was complicated and the reconstruction was time-consuming [24]. You introduced a smart active optical sensor, in which a composite pattern was utilized. However, monocular vision uses a single feature point for measurement, which is easy to produce errors due to the inaccurate feature point extraction [25]. Sun proposed a sensor for in-motion continuous 3D shape measurement based on dual line-scan cameras, but the sensor was only verified with a uniform linear motion [26].

In view of the above problems, a structured light three-dimensional measurement system with a low-cost was developed and verified in this paper. In the developed system, three fringe patterns with proper wavelengths were projected on the object and the wrapped phase was obtained using the three wavelengths directly, i.e., the calculations of the equivalent wavelengths and their corresponding phase maps were not needed, which not only greatly reduces measuring time, but also improves its ability to distinguish the details of the shape. In addition, it does not cause color damage to cultural relics and can overcome the shortcoming of weak reflectivity of black objects in laser scanning. In terms of cost, it is much lower than the laser 3D scanner as ordinary CCDs (Charge-Coupled Device) and projectors are used to implement the system.

This paper is organized as follows. In Section 2, we introduce the three-dimensional measurement method based on three wavelength grating projection. In Section 3, by taking Terra-Cotta Warriors as examples, three-dimensional measurements were conducted to generate three-dimensional models, which were used for packaging. In Section 4, we evaluate the accuracy of the proposed method. Finally, Section 5 concludes this paper.

## 2. Three-Dimensional Measurement Based on Three Wavelength Grating Projection

To avoid damage to cultural relics during transportation, accurate point cloud data needs to be acquired through three-dimensional measurement for packaging. We have developed a three wavelength grating projection method. Compared to the traditional multi-frequency heterodyne method, based on synthetic phases [27,28], our method uses the phase information of three different wavelengths for phase unwrapping and phase correction, which saves the synthetic phase and can obtain more accurate point cloud data, facilitating the following three-dimensional modeling. The method is detailed in the following.

The grating stripes with wavelengths $\lambda_{1}=1008$ pixels, $\lambda_{2}=144$ and pixels, and $\lambda_{3}=16$ pixels, and pixels are used for projection. Each pixel point, (x,y), experiences six projections, with phase shift amounts of 0, 2π/6, 4π/6, π, 8π/6 and 10π/6 [29]. The gray values of the point are denoted by $I_{1}(x,y)$, $I_{2}(x,y)$, $I_{3}(x,y)$, $I_{4}(x,y)$, $I_{5}(x,y)$, and $I_{6}(x,y)$. The wrapped phase, $\varphi_{i}(x,y)$, corresponding to $\lambda_{i}$ (*i* = 1, 2, 3) was obtained as

(1) $$\varphi_{i}(x,y)=\tan^{-1}\left(\frac{I_{3}(x,y)-I_{5}(x,y)}{I_{4}(x,y)-I_{1}(x,y)+I_{3}(x,y)-I_{5}(x,y)}\right).$$

The unwrapped phase, $\Phi_{3}(x,y)$, was calculated as

(2) $$\Phi_{3}(x,y)=\varphi_{3}(x,y)+2\pi \times \left\{\mathrm{INT}\left(\frac{\left(\varphi_{3}(x,y\right)}{2\pi }\times N_{1}\right)\times N_{2}+\mathrm{INT}\left(\frac{\left(\varphi_{2}(x,y\right)}{2\pi }\times N_{2}\right)\right\}$$

where INT returns the value of a number rounded upward to the nearest integer. When the phase, $\varphi_{3}(x,y)$, is near 2π, a phase jump may occur so the phase unwrapping operation in Equation (2) needs to be corrected. When $\varphi_{1}(x,y)<2\pi -\delta$ or $\varphi_{1}(x,y)>2\pi +\delta$, and $\varphi_{2}(x,y)<2\pi -\delta$ or $\varphi_{2}(x,y)>2\pi +\delta$, and $\varphi_{3}(x,y)<2\pi -\delta$ or $\varphi_{3}(x,y)>2\pi +\delta$, the unwrapped phase, $\Phi_{3}(x,y)$, was corrected as

(3) $$\Phi_{3}(x,y)=\varphi_{3}(x,y)+2\pi \times \left\{\mathrm{INT}\left(\frac{\left(\varphi_{1}(x,y\right)}{2\pi }\times N_{1}\right)\times N_{2}+\mathrm{INT}\left(\frac{\left(\varphi_{2}(x,y\right)}{2\pi }\times N_{2}\right)\right\},$$

where $N_{1}=\lambda_{1}/\lambda_{2}$, $N_{2}=\lambda_{2}/\lambda_{3}$, and δ is a decimal number approaching 0. When $\varphi_{1}(x,y)<2\pi -\delta$ or $\varphi_{1}(x,y)>2\pi +\delta$, and $2\pi -\delta <\varphi_{2}(x,y)<2\pi +\delta$, or $\varphi_{3}(x,y)<2\pi -\delta$, or $\varphi_{3}(x,y)>2\pi +\delta$, the unwrapped phase, $\Phi_{3}(x,y)$, is corrected as

(4) $$\Phi_{3}(x,y)=\varphi_{3}(x,y)+2\pi \times \left\{\mathrm{INT}\left(\frac{\left(\varphi_{1}(x,y\right)}{2\pi }\times N_{1}\right)\times N_{2}+\mathrm{INT}\left(\frac{\left(\varphi_{2}(x,y\right)}{2\pi }\times N_{2}\right)-1\right\}.$$

When $2\pi -\delta <\varphi_{1}(x,y)<2\pi +\delta$, and $\varphi_{1}(x,y)<2\pi -\delta$ or $\varphi_{1}(x,y)>2\pi +\delta$, and $\varphi_{3}(x,y)<2\pi -\delta$ or $\varphi_{3}(x,y)>2\pi +\delta$, the unwrapped phase, $\Phi_{3}(x,y)$, was corrected as

(5) $$\Phi_{3}(x,y)=\varphi_{3}(x,y)+2\pi \times \left\{\mathrm{INT}\left(\frac{\left(\varphi_{1}(x,y\right)}{2\pi }\times N_{1}-1\right)\times N_{2}+\mathrm{INT}\left(\frac{\left(\varphi_{2}(x,y\right)}{2\pi }\times N_{2}\right)\right\}.$$

## 3. Three-Dimensional Reconstruction

Three-dimensional reconstruction is based on 3D scanning and the generated 3D point cloud data were processed to obtain a digitized 3D model. Due to the large size of the heritage models and the limited scanning scope of the scanner, the measurements needed to be spliced after partial scanning. The main steps of the 3D reconstruction included point cloud preprocessing, registration, merging, thinning and grid reconstruction.

### 3.1. Point cloud preprocessing

**Figure 1:**
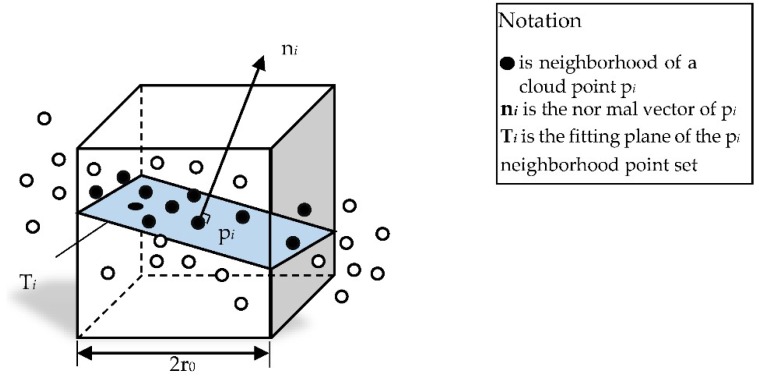
Neighborhood searching.

**Figure 2:**
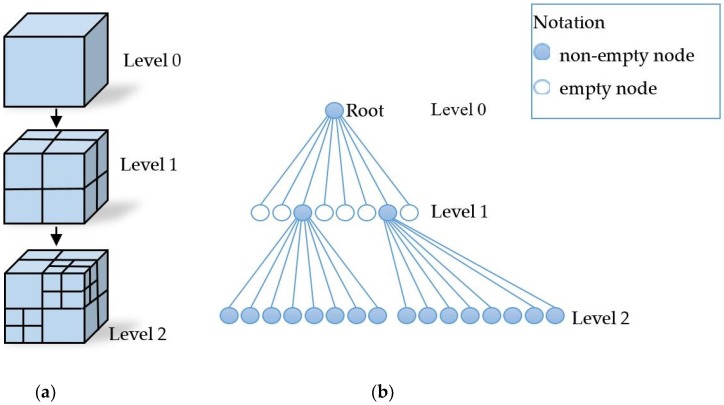
Octree. (**a**) Space decomposition, (**b**) Octree hierarchical structure.

The scanning point cloud usually has defects, e.g., due to external noise, pre-processing was needed before subsequent processing. The point cloud processing required neighborhood information of the point cloud [30]. Figure 1 shows the neighborhood of a cloud point p*_i_* and the corresponding normal vector and fitting plane. The neighborhood searching methods were well studied and we refer readers to Reference [31] for the details. The octree structure [32] was used to speed up the neighborhood search, as shown in Figure 2. In order to improve the efficiency, the octree building and even neighborhood point search were completed when the point cloud was imported. Search the neighborhood to synthesize different types of surfaces and perform denoising according to the point cloud requirements.

### 3.2. Registration

**Figure 3:**
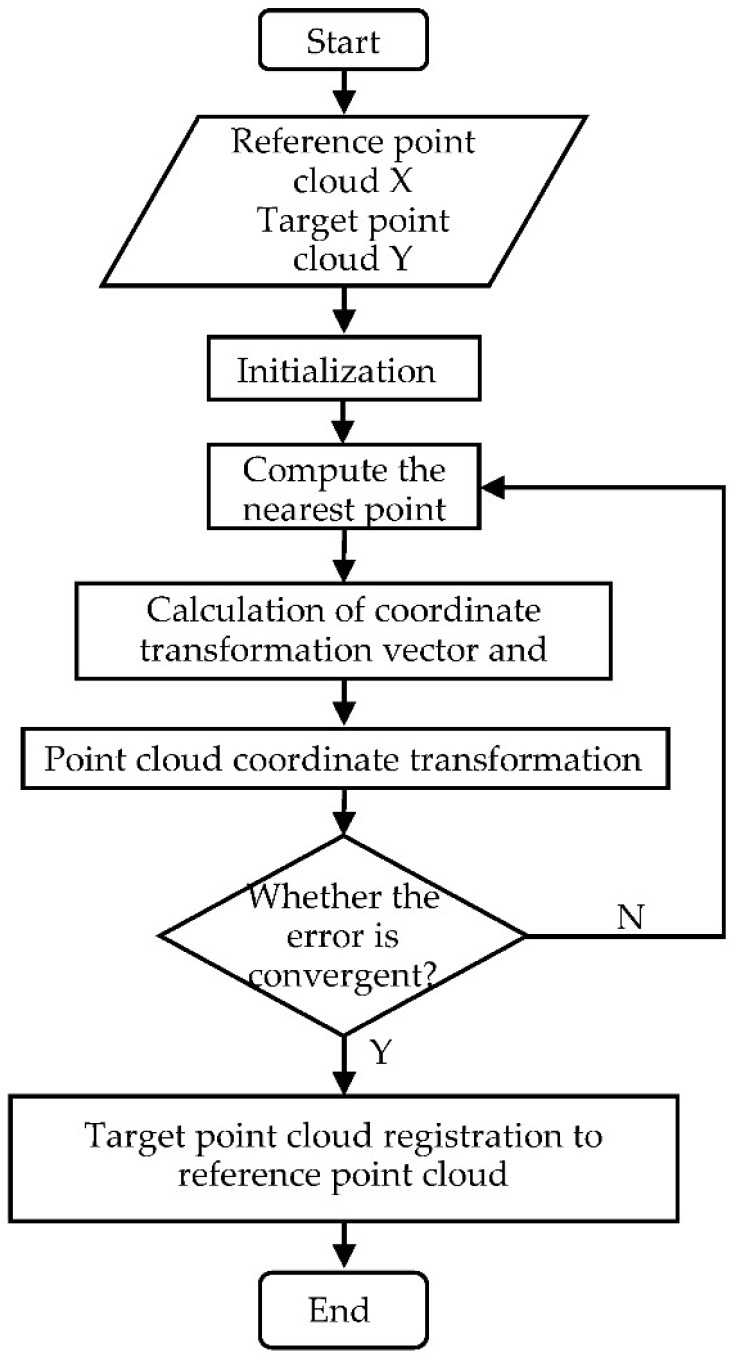
ICP algorithm.

In the scanning process of the Terra-Cotta Warriors, the point cloud data obtained by the scanner may have translation dislocation and rotation dislocation. In order to obtain accurate and complete point cloud data, registration of the obtained local point cloud data was needed. In this work, the ICP (Iterative Closest Point) algorithm [33,34] was used to make it repeat the process of determining the set of corresponding relation points and calculating the optimal convergence, until a convergence criterion for the correct matching was satisfied so that it is converted to a unified coordinate system. Its principle flowchart is shown in Figure 3.

### 3.3. Merging

Different cloud spots will appear in the public area. Although the point clouds in these public areas after registration have been consistent, they will not coincide completely. In order to reintegrate the dispersed point cloud into a whole, data merging was needed. The merging of point clouds in public areas can avoid the error of surface reconstruction due to point cloud stratification.

### 3.4. Thinning

Due to the large size of the Terracotta Army, the volume of data obtained by scanning is very large. A large volume of data is beneficial to improve the registration and merging accuracy, but it is a burden for the subsequent grid reconstruction. While the computation increases, the accuracy of the mesh reconstruction may not be improved, but the precision is reduced because of overfitting. Therefore, under the premise of guaranteeing accuracy, the point cloud was thinned and the point cloud after descending sampling was used for subsequent processing.

### 3.5. Grid reconstruction

The surface of the real object was continuous, but the data obtained from the point cloud were discrete and cannot be simulated accurately. Therefore, grid reconstruction was needed to mesh the grid model of point clouds. There are many methods for mesh reconstruction. The triangulation grid is usually used. This grid computing method is simple and easy to handle and the generated model is adaptive.

## 4. Experimental Section

**Figure 4:**
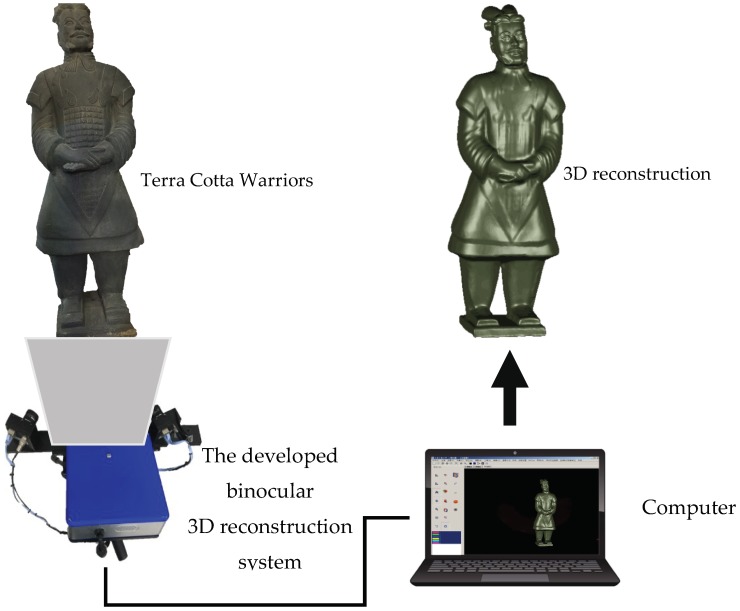
The binocular 3D reconstruction system developed in our Lab.

A three-dimensional measurement and reconstruction system for cultural relics was developed in our lab, including both software and hardware. The developed binocular 3D reconstruction system is shown in Figure 4, where an LG (HX300G) projector with a resolution of 1024 × 768 was used to cast three frequency cosine white stripes and the three cosine photoperiods were 1008 pixels, 144 pixels, and 16 pixels, respectively. The light source of the projector was non-interference light with a wavelength of 450–465 nm. The industrial camera RS-A1300-GM60 (manufactured by the Beijing Microview Science and Technology Co. Ltd., Beijing, China) sent a 1280 × 1024 gray image to the computer at a frame rate of 4 frames/s. The industrial camera used an 8 mm (M0814-MP2) lens. The system was about 500 mm away from the Terra Cotta Warrior and the angle between the two cameras was about 60°. The binocular 3D reconstruction system used two grayscale cameras, i.e., the grayscale image was captured through a single channel. The R, G, B trichromatic light was projected in order and the actual color of the measured object was obtained through color difference synthesis of the three colors. In this way, color point cloud data were obtained and the processing speed was also faster compared to the processing of three channel color images, which also helped to reduce the cost to some extent.

**Figure 5:**
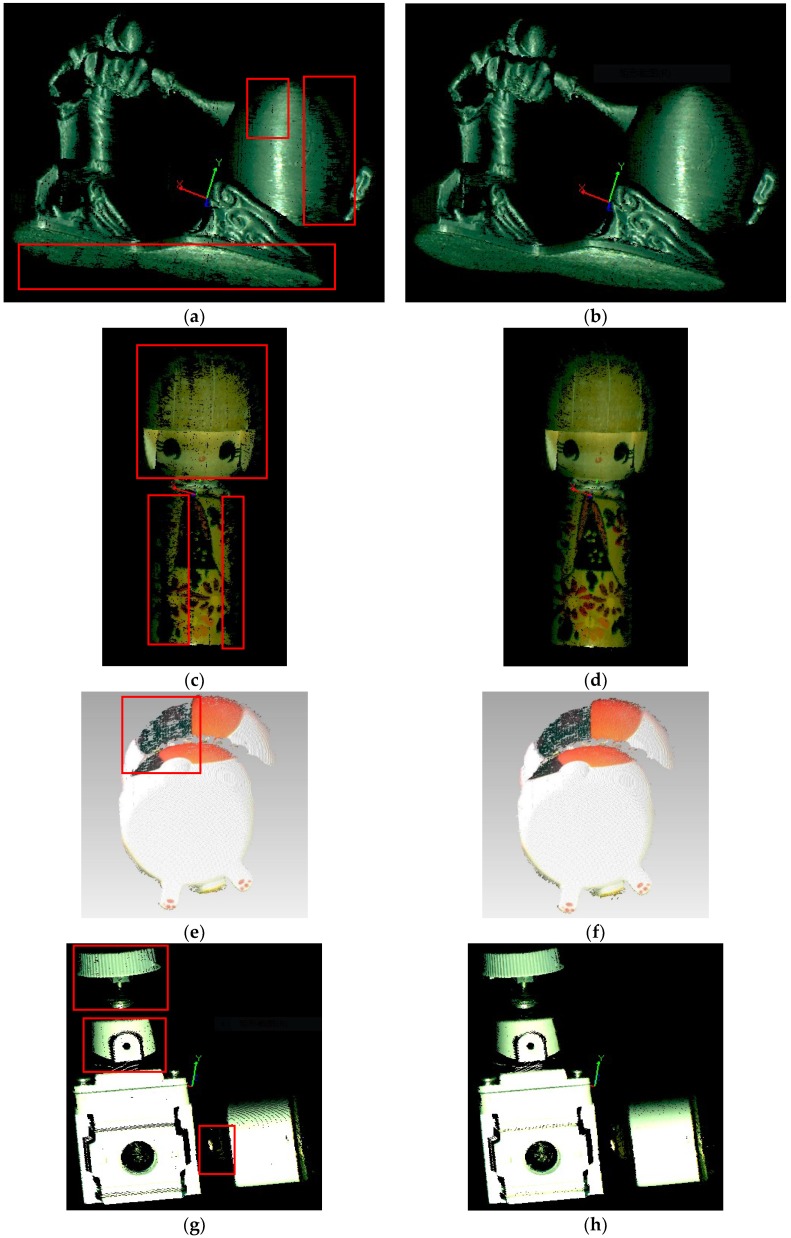

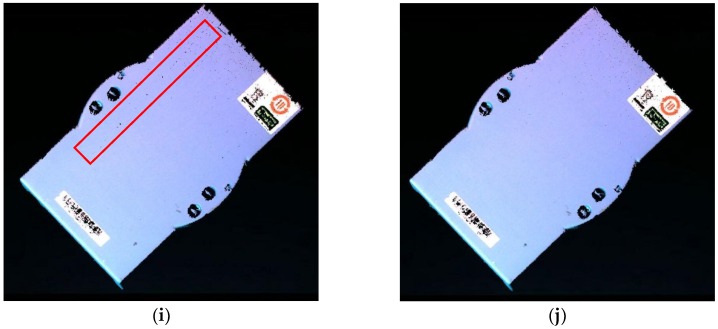
Comparison of the reconstruction results. (**a**) The traditional method (grey anime dolls). (**b**) The proposed method (grey anime dolls). (**c**) The traditional method (colored puppets). (**d**) The proposed method (colored puppets). (**e**) The traditional method (colorful kittens). (**f**) The proposed method (colorful kittens). (**g**) The traditional method (air valves). (**h**) The proposed method (air valves). (**i**) The traditional method (flat). (**j**) The proposed method (flat).

We compared our method with the multi-frequency heterodyne method [35] in the experiments. Three-dimensional measurements of grey anime dolls, colored puppets, colorful kittens, air valves, and flat were carried out and the experiment results are shown in Figure 5. The parts within the red rectangles in the picture show the differences between the two methods, where we can see that the traditional method exhibits data loss towards the edges the point cloud and the proposed method is more accurate.

**Figure 6:**
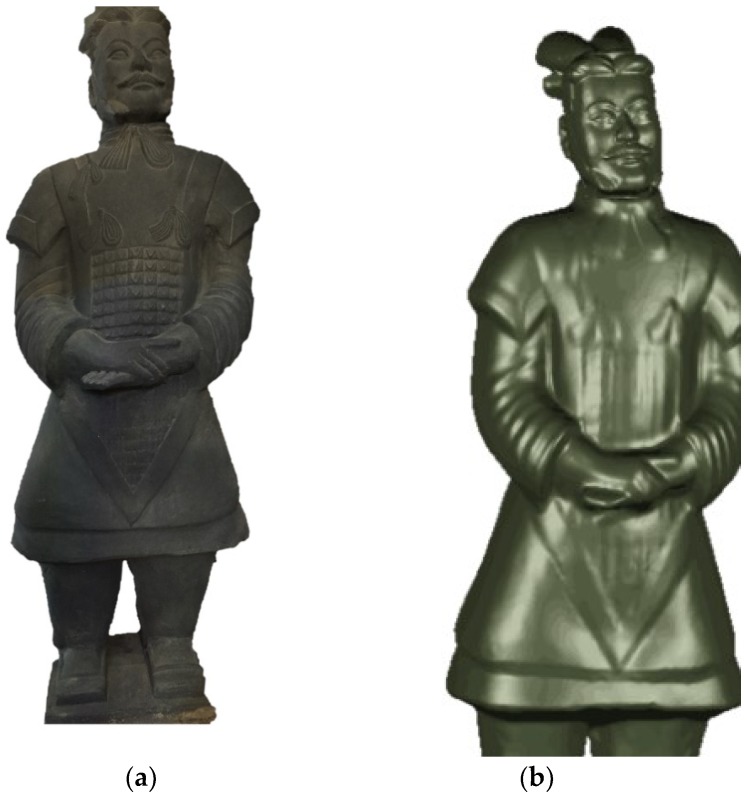
Field map. (**a**) Terra Cotta Warrior; (**b**) 3D reconstruction.

**Figure 7:**
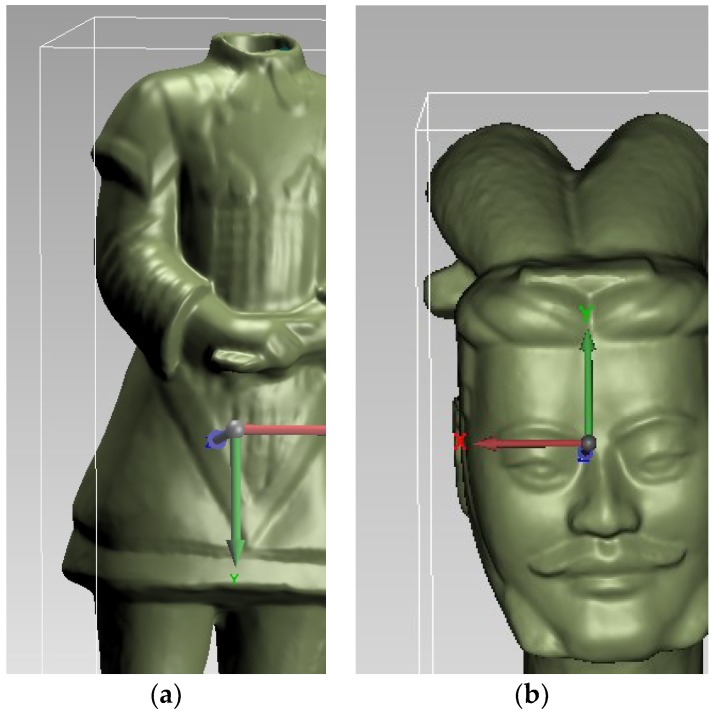
The results of the 3D reconstruction. (**a**) Body (**b**) Head.

Shown in Figure 6 is a site survey map of Terra-Cotta Warriors. The Terra-Cotta Warriors were divided into head and body parts, which are disassembled during transportation. The 3D measurement and reconstruction experiments of Terra-Cotta Warriors were carried out by using our system and Figure 7 shows the 3D reconstruction results.

The experimental results show that the method is efficient, accurate and reliable. Table 1 lists the results of the calibration of the system using a high precision plane calibration board.

**Figure 8:**
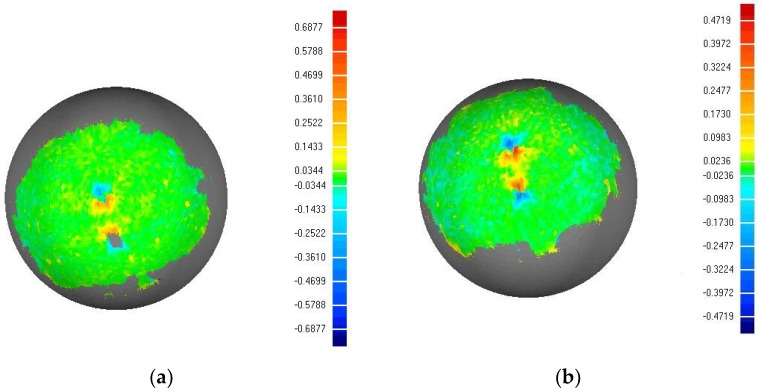
Geomagic studio sphere fitting comparison. (**a**) The fitting result of the traditional method (**b**) The result of the method in this paper.

To further examine the performance of the proposed method, we used the traditional phase unwrapping method and the method in this paper to measure a calibrated sphere with a diameter of 20 mm. The accuracy of the measurement reached 0.0001 mm. A 3D sphere model with a diameter of 20 (0.0001) mm was created and the calibration ball 3D point cloud data, obtained by two methods, were fitted to the 3D data of the standard sphere by Geomagic studio. The comparison diagram is shown in Figure 8 and the comparison results are listed in Table 2. It can be seen that the relative precision of the system reached a level of 1/1000. As we can see from Figure 8, the 3D point cloud data obtained by the traditional method has some holes in the point cloud. The point cloud data obtained by this method were denser and the data were more accurate. As shown in Table 2, the method is better than the traditional method in terms of the average distance, standard deviation and the root mean square, which fully demonstrates the superiority of the method.

The Terra-Cotta Warriors were divided into head and body parts, which are disassembled during transportation. The scene personnel provided the size of the Terra-Cotta Warriors as follows: The actual length of the body part is 40 cm; the width is 39.6 cm; the height is 94.4 cm; the actual length of the head is 27.1 cm; the width is 29 cm; and the height is 17.2 cm, which are listed in Table 3. Table 4 compares the measurements and the actual size of the Terra-Cotta Warriors. We measured the distance between any two features of the Terra-Cotta Warriors by Geomagic studio and compared them to the actual value. For example, the distance between the corners of the two eyes, the distance between the two corners of the mouth and the distance between the two feet, which are listed in Table 5. The experimental results show that the accuracy of the system for measuring large objects is about 0.1 mm (due to the post-processing of point clouds), which meets the packaging requirements of cultural relics to effectively protect the cultural relics during transportation.

**Figure 9:**
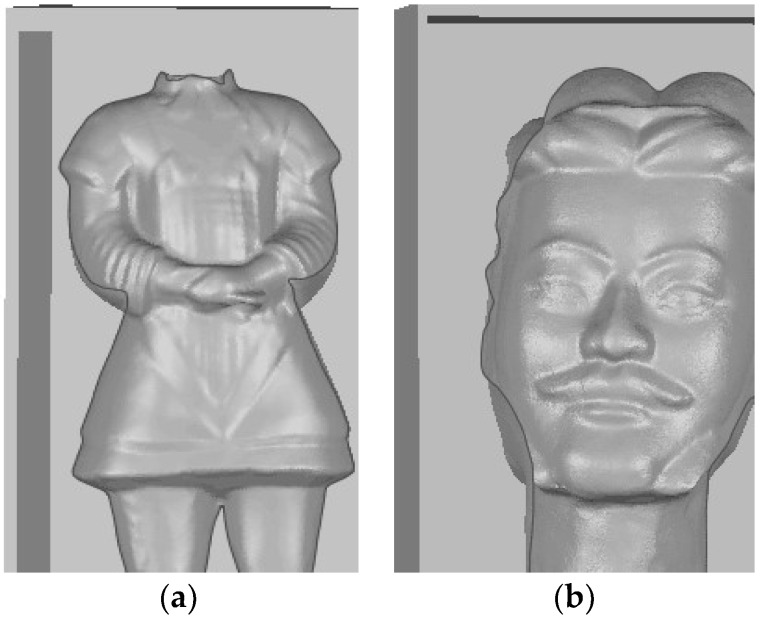
A sample of the packing for transportation. (**a**) Body, (**b**) Head.

Shown in Figure 9 is a sample of the packing for transportation, where the original size data obtained from the three-dimensional reconstruction were used for mold processing.

## 5. Conclusions

In this work, we have developed a structure light-based three-dimensional reconstruction system for cultural relic packaging. Compared to the laser scanning based equipment, the one developed in this work had several advantages. The laser scanning equipment is expensive, and it involves complex field operations and data processing (e.g., to obtain the texture of the object). In contrast, the developed system has a low-cost as it only requires two ordinary industrial cameras, one computer and a projector. Moreover, the developed system requires simpler field operations (capturing pictures with cameras and requiring less in-site brightness control). The point clouds and color textures were automatically generated by using our developed software and the accuracy reached 1/1000. In addition, the reconstruction process was fast, e.g., it took about 13 s in our experiments. Besides cultural relic packaging, the three-dimensional models obtained by our system can be used for museum display, which enables the interactive display of cultural relics.

Our future work includes adding a turntable to the system to achieve self-splicing and further research on improving the measurement precision in large scenes. Another possible future work is to build a digital three-dimensional cultural relic model database, through a cultural relics management system.

## Figures and Tables

**Table 1:** Calibration results for the plane calibration board with different distances.

Scanning Distance (cm)	Absolute Mean Value of Error (mm)	General Standard Deviation (mm)
30	0.0227	0.0212
40	0.0229	0.0212
50	0.0300	0.0303
60	0.0336	0.0343

**Table 2:** Data comparison of fitting results.

	Average Distance (mm)	Standard Deviation (mm)	Root Mean Square (mm)
The traditional method	0.0115	0.0501	0.0514
The method in this paper	0.0016	0.0493	0.0493

**Table 3:** Comparison of the three-dimensional reconstruction measurements of body parts and the actual size of the Terra-Cotta Warriors.

Three-Dimensional Measurement Boundary Frame (mm)	Dimensions of Three-Dimensional Measurement (mm)	Actual Size of an Object (mm)	Error (mm)
X [−135.6255, 265.2018]	400.8273	400.0	0.8273
Y [−209.0496, 734.4292]	943.4788	943.5	0.0212
Z [−8.7729, 387.0705]	395.8434	396.0	0.1566

**Table 4:** Comparison of measurement dimensions of head 3D reconstructions and actual size of the Terra-Cotta Warriors.

Three-Dimensional Measurement Boundary Frame (mm)	Dimensions of Three-Dimensional Measurement (mm)	Actual Size of an Object (mm)	Error (mm)
X [−64.2971, 207.2918]	271.5889	271.0	0.5889
Y [−25.8170, 264.2313]	290.0483	290.0	0.0483
Z [−108.8357, 63.6009]	172.4366	172.0	0.4366

**Table 5:** 3D measurement data and actual value.

X Axis (mm)	Y Axis (mm)	Z Axis (mm)	3D Data (mm)	Actual Value (mm)	Error (mm)
78.081	5.441	3.291	78.340	78.501	0.161
37.899	3.279	0.494	38.044	38.000	0.044
223.785	2.242	10.705	224.052	224.00	0.052
